# Super-Resolution Reconstruction of Cytoskeleton Image Based on A-Net Deep Learning Network

**DOI:** 10.3390/mi13091515

**Published:** 2022-09-13

**Authors:** Qian Chen, Haoxin Bai, Bingchen Che, Tianyun Zhao, Ce Zhang, Kaige Wang, Jintao Bai, Wei Zhao

**Affiliations:** 1 School of Automation, Northwestern Polytechnical University, Xi’an 710129, China; 2 State Key Laboratory of Photon-Technology in Western China Energy, International Collaborative Center on Photoelectric Technology and Nano Functional Materials, Institute of Photonics & Photon Technology, Northwestern University, Xi’an 710127, China

**Keywords:** super-resolution reconstruction, A-net, deep learning network, cytoskeleton

## Abstract

To date, live-cell imaging at the nanometer scale remains challenging. Even though super-resolution microscopy methods have enabled visualization of sub-cellular structures below the optical resolution limit, the spatial resolution is still far from enough for the structural reconstruction of biomolecules in vivo (i.e., ~24 nm thickness of microtubule fiber). In this study, a deep learning network named A-net was developed and shows that the resolution of cytoskeleton images captured by a confocal microscope can be significantly improved by combining the A-net deep learning network with the DWDC algorithm based on a degradation model. Utilizing the DWDC algorithm to construct new datasets and taking advantage of A-net neural network’s features (i.e., considerably fewer layers and relatively small dataset), the noise and flocculent structures which originally interfere with the cellular structure in the raw image are significantly removed, with the spatial resolution improved by a factor of 10. The investigation shows a universal approach for exacting structural details of biomolecules, cells and organs from low-resolution images.

## 1. Introduction

Microscale organizations and nanoscale biomolecular structures play essential roles in life machinery, e.g., the nanopores control transportation [1] and the cytoskeleton behaves as a mechanosensor [2]. To understand the underlying mechanism of cellular behavior, it is important to monitor the dynamics of biomolecules at resolution of tens of nanometers, e.g., the ~50 nm persistence length of DNA [3] and ~24 nm thickness of microtubule fiber [4]. Imaging platforms, which are reported to achieve such resolution, include transmission electron microscopy (TEM, 300 nm) [5], scanning electron microscopy (SEM, 200 nm) [6], cryogenic electron microscopy (Cryo-EM, 200 nm) [7] and stimulated emission depletion (STED, 20 nm) microscopy [8], etc. TEM, SEM and Cyto-EM, however, are not suitable for live-cell imaging and monitoring molecular dynamics in vivo [9]. STED microscopy is a promising technique. Its application is, however, hindered by the presence of specific fluorophores [10], excessively complex operational procedures [11], and high cost [12]. Considering the fact that there exist large quantities of image data in various databases [13], and most laboratories are only equipped with commonplace inverted microscopes with sub-micron resolution and high noise level, it is critical to develop a numerical approach, which can exact molecular information from poor quality images.

Currently, reported image processing algorithms can be categorized as traditional [14,15], and deep-learning image processing algorithms. The latter have become a focus of the image processing community, and many algorithms have been developed. For instance, the super-resolution convolutional neural network (SRCNN) [16] is an end-to-end network developed and based on sparse coding to obtain a sharper edge and higher resolution of images. The shortcomings of SRCNN include the sacrifice of processing speed to realize an acceptable restoration quality. The fast super-resolution convolutional neural network (FSRCNN) [17] is an update of SRCNN. It provides a large speed boost, while simultaneously losing details as a result of excessive smoothing. The super-resolution generative adversarial network (SRGAN) [18] optimizes loss function to obtain high PSNR (peak SNR) and to enhance the restored image’s sense of reality. Visually, the restored images show better reality; however, the PSNR of images is reduced. Additionally, these algorithms primarily aim to enrich the pixel information of the image and not to improve the image’s intrinsic optical resolution.

In this investigation, a deep learning network named A-net is proposed by improving the structure of the U-net network. Accompanied with a traditional degradation model to process label images, the details of a microtubule network captured by a confocal microscope can be extracted with higher resolution and SNR. In brief, raw images were firstly processed by threshold denoising and a three-dimensional Gaussian interpolation. Then, the corresponding label images were obtained using the DWDC method [19], which combines discrete wavelet and Lucy–Richardson deconvolution [20] to extract finer structures. The pairs of original images with the corresponding label images served as our own datasets, relying on which the A-net network was trained. Finally, the test images were processed according to the A-net network. It is demonstrated that our method can effectively remove noise and flocculent structures in the raw images, resulting in ~10 times increased resolution.

## 2. Related Works

As the purpose of this paper is to explore super-resolution algorithms based on neural networks, the existing algorithms for improving image resolution are reviewed in the first part, followed by detailed introduction on the super-resolution algorithms based on deep learning.

### 2.1. Traditional Methods

Traditional image processing algorithms mainly rely on basic digital image processing techniques. Generally, there are three categories: interpolation-based algorithms [21,22,23], degenerate-model-based algorithms [24,25,26] and learning-based algorithms [27,28,29,30].

Interpolation-based algorithms [31,32] use the original pixel information of the low-resolution image to “guess” the sub-pixel information of image based on interpolation. It can effectively upgrade the low-resolution image to high resolution with more pixels. Nevertheless, in practical applications, the interpolation algorithms can only improve the image details in a very limited way.

Degenerate-model-based algorithms [33,34] focus on establishing an observation model for the acquisition process of images, and then realize super-resolution reconstruction by solving the inverse problem of the observation model. The observation model describes the process of obtaining the low-resolution observation image from the high-resolution image by the imaging system, as shown in Formula (1):

(1) L=H∗f+N

where *L* is the low-resolution image, *H* is the high-resolution image, f is a transformation function (i.e., the point-spread function in the optical system) and N is noise. This method restores the actual information of the object with a higher resolution, based on the estimation of f. Commonly, this type of super-resolution algorithm includes iterative backprojection (IBP) [34], projection of convex set (POCS) [25], maximum posterior probability (MAP) [35], or Bayesian analysis [36,37] methods, etc. These methods aim improve the visual quality of images and restore object details. However, they also suffer a series of problems, e.g., the processing speed is generally slow and may lead to spurious images.

Learning-based algorithms [38,39] aim to build a mapping between the low-resolution image and the corresponding high-resolution image by prior training and learning from the dataset. Learning-based algorithms are mainly realized by machine learning. There are several commonly used machine learning methods, including neighborhood embedding [40], support vector regression [41,42], manifold learning [43], sparse representation [44,45], etc. Learning-based algorithms are limited by several disadvantages including the need to manually optimize parameters and the lack of end-to-end training, which leads to poor algorithm applicability.

### 2.2. Deep-Learning-Based Algorithm

In recent years, various deep-learning-based super-resolution algorithms have been developed. Dong et al. [16] first applied a deep neural network to super-resolution processing. They proposed SRCNN to learn the end-to-end mapping between low-resolution images and corresponding high-resolution images. A three-layer convolutional neural network is combined with bilinear interpolation and nonlinear mapping to form the SRCNN algorithm. SRCNN algorithm automatically optimizes all parameters by learning from the input training set, and can therefore reach an average PSNR value of 30.09 dB with a runtime of 0.18 s per image. Dong et al. [17] developed FSRCNN based on SRCNN. A deconvolution layer was used at the end of the network to enlarge the image size, which can save time by eliminating the pretraining phase. The network also replaces convolution kernels in SRCNN with smaller convolution kernels and shares convolution layers in order to reduce the computation. These improvements help the network reduce the calculation parameters and speed up the processing. As a consequence, FSRCNN has very fast processing speed without the loss of restoration quality and achieves an average PSNR of 32.87 dB with a processing speed of 24.7 fps.

Kim et al. [46] extended the network to 20 layers based on SRCNN and introduced residual structure into the network, i.e., very deep convolutional networks (VDSR). The deep network layer has a larger receptive field, and more information can be learned with better accuracy. The VDSR network uses the residual learning method to limit the gradient to a certain range, which can speed up the convergence process. As compared to SRCNN, the VDSR network realized higher accuracy, faster convergence, and greater resolution of folds. In their investigation, VDSR achieves an average PSNR of 37.53 dB.

In the aforementioned methods, excessive smoothing of an image is inevitable and could lead to a spurious image. Ledig et al. [18] proposed a generative adversarial network (SRGAN), which is developed on the basis of the generative adversarial network (GAN) to solve the problem for super-resolution processing and recovering finer texture structures. The generate network generates high-resolution prediction images from low-resolution original images, and the discriminate network determines whether the prediction image is consistent with the corresponding label image. Although the PSNR values were not apparently improved, the details of the image were enhanced to super-resolution level. It should be noted, again, they aim to enrich the pixel information of the image, e.g., from 512 × 512 pixels to 1024 × 1024 pixels, not to improve the intrinsic optical resolution of image, e.g., from 300 to 100 nm.

## 3. Algorithm

**Figure 1:**
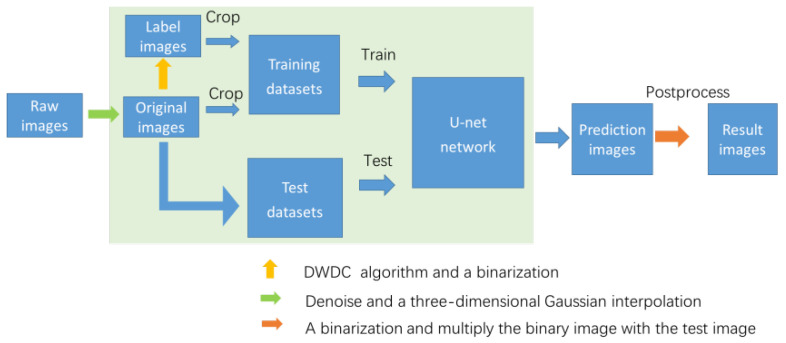
Overall framework of the algorithm. A series of preprocessing methods are used to obtain the original image and the label image from the raw image. Denoise and a three-dimensional Gaussian interpolation is performed on the raw confocal images to obtain the original image, as shown by the green arrow. Then, the DWDC algorithm and a binarization are used to obtain high-resolution label images (shown by the yellow arrow). The original image and the corresponding label image are composed of image pairs and cropped into 512 × 512 size to construct the biological microtubule image dataset, referred to as the SR_MUI dataset. A-net network trains the parameters in the network through the image of the training dataset, and then obtains the corresponding prediction results. In postprocessing, as shown by the orange arrow, applying binarization on the prediction image obtains a shrinkage outline of the microtubule structures. Subsequently, the binary image is multiplied by the test image to acquire the result image.

To improve the resolution of a poor quality image intrinsically, i.e., extract the real structure from a blurred and noisy image, a new algorithm combining traditional image preprocessing algorithms based on the degenerate model and A-net network [47] is proposed here. The overall framework of this algorithm is shown schematically in Figure 1. For the A-net deep learning network, the image pairs of original and label images are required to construct training datasets for the deep neural network. Because of the scarcity and particularity of biological images, it is necessary to build our own biological microtubule image dataset (i.e., SR_MUI dataset), which is obtained using the DWDC method [19] that constitutes of a series of preprocessing methods, discrete wavelet method, Lucy–Richardson deconvolution method and postprocessing methods. The training dataset is input into the network so that the A-net network can learn the mapping relationship between low-resolution images and high-resolution label images. The test dataset is then input into the A-net network for prediction, and the super-resolution images are obtained.

### 3.1. Raw Images and Processing Targets

**Figure 2:**
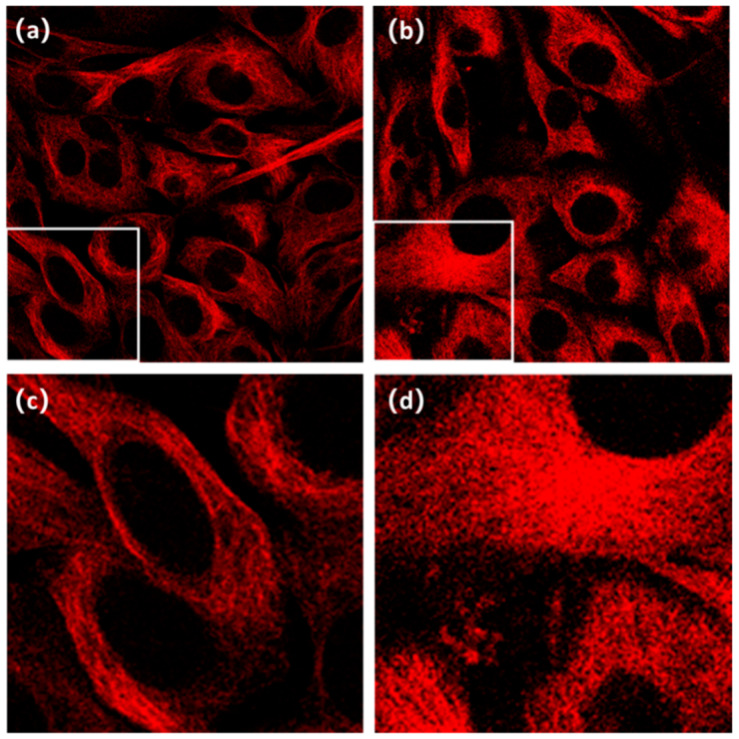
Raw images captured by confocal microscope. The raw images are 3t3 cell microtubule images captured by a commercial confocal microscope (Nikon A1 LFOV) with an Olympus 100X NA1.4 oil immersion objective lens. Images in (**a**,**b**) show the different structures and shapes; (**c**,**d**) show separately the details of local regions within the white boxes of (**a**,**b**). The raw images are all of 512 × 512 size.

The raw images to be processed in this paper are confocal fluorescent images of 3T3 fibroblast microtubule labeled by tubulin fluorescent dye, which is excited at 640 nm and emitted around 674 nm (Figure 2). The raw images were captured by a commercial confocal microscope (Nikon A1 LFOV) using an Olympus 100X NA1.4 oil immersion objective lens. Each raw confocal image is in 16-bit TIF format with a size that is 512 × 512 pixels. The pixel interval is 0.25 µm.

It can be observed that the filament-like microtubule structures are widely present in cells, characterized by poor SNR, insufficient spatial resolution and much noise information. In the investigation, it is our goal to develop a universal algorithm to obtain super-resolution images of microtubules from such low-resolution images.

### 3.2. Preprocessing

In order to improve the SNR of the image, threshold denoising is used first to reduce image noise. Since the pixel interval in the raw image is 0.25 µm, it restricts the image resolution to be further improved. To restore the details of the targets, a three-dimensional Gaussian interpolation is performed twice, with the Gaussian function as $g=g_{0}\exp\left[-\frac{{\left(x-x_{c}\right)}^{2}+{\left(y-y_{c}\right)}^{2}}{2r_{⊥}^{2}}-\frac{{\left(z-z_{c}\right)}^{2}}{2r_{∥}^{2}}\right]$*,* where $r_{⊥}=0.61\lambda_{e}/N_{A}$ and $r_{∥}=4n\lambda_{e}/2N_{A}^{2}$ being the transverse and axial radius, respectively; $x_{c}$, $y_{c}$ and $z_{c}$ are the interpolation center coordinates; n is the refraction index of medium; $N_{A}$ is the numerical aperture of the lens and $\lambda_{e}$ is the wavelength of the excitation beam.

Accordingly, the image size is extended from 512 × 512 to 2048 × 2048, leading to reduced pixel interval of 63 nm. The z-stack interval is also reduced from 1 μm to 250 nm. Then, the DWDC algorithm is used to obtain high-resolution label images [19]. In this method, discrete wavelet analysis and the Lucy–Richardson deconvolution method are combined, with binarization and threshold processing, in order to extract the sketch of microtubule structures and prevent the detailed information to be immersed by the background. The method is capable of significantly improving the image resolution of a 3t3 fibroblast microtubule up to 15 times and realizes 123.7 nm resolution. The details of the structure of the microtubule are clearly reserved. Therefore, it is appropriate to use the DWDC method to obtain the label images.

It is well known that larger image size increases the number of network parameters, e.g., the size of the convolutional layer and the computation cost of the loss function. In the process, the expansion brings heavy burden to the server and network for computing. For instance, if the size of the feature convolution layer according to a 512 × 512 image imported into the U-net model is 32 × 32 × 1024, then the size of that when importing a 2048 × 2048 image into the U-net model is 128 × 128 × 1024. The storage requirement is increased 16 times and the computation cost could be more than 16 times, since the neural network is nonlinear.

**Figure 3:**
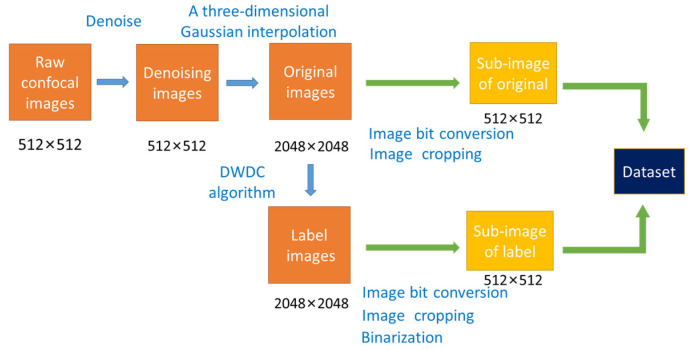
Diagram of the preprocessing procedures. At the beginning, both threshold denoising and a three-dimensional Gaussian interpolation are carried out on the raw confocal images. The image after these processing steps is adopted as the original image for the A-net network. Then, the DWDC algorithm is applied on the original images to obtain high-resolution label images. The label image is further binarized to prevent the network from learning additional feature information. Subsequently, both the original and label images are converted from 16-bit data to 8-bit, and split from 2048 × 2048 pixels to 16 sub-images of 512 × 512 pixels, to reduce the load of A-net computation. Finally, the corresponding sub-image pairs form the SR_MUI dataset.

To improve the efficiency of training, a series of preprocessing steps are made, as diagrammed in Figure 3. On one hand, the original 16-bit TIF images are converted to 8-bit TIF images by projecting data from 16-bit to 8-bit using an approximately linear approach. On the other hand, each image of 2048 × 2048 size, either the original one or the label one, is split into 16 sub-images of 512 × 512 size. Thus, the SR_MUI dataset is constructed by pairing the sub-images corresponding to the original and label images.

It is worth mentioning that since the image size of the training set is 512 × 512, when the test image is input into the A-net network, each test image needs to be divided into 16 sub-images of 512 × 512 pixels as well. These sub-images of test images are processed by the A-net network and the corresponding super-resolution prediction images are obtained.

### 3.3. A-Net Network

This investigation focuses on the filament-like microtubule structures that can be approximated as a cluster or mesh of segments. Thus, the U-net network, which has been widely used in image segmentation, was applied here for biostructure extraction and super-resolution processing. One of the most significant advantages of the U-net is that it does not require a large biological dataset. This is particularly important for us, since our dataset is relatively small and there are no established works or public datasets that can fulfill our purpose.

The U-net network is composed of the encoder network and the decoder network with symmetric structures. In the encoder network, there are four convolution blocks for feature maps of different sizes. In the convolution block, there are two 3 × 3 convolutions in sequence, followed by 2 × 2 max pooling. In the decoder network, there are also four deconvolution blocks corresponding to the encoder network. In the deconvolution block, there are two 3 × 3 convolutions in sequence, followed by a transposed convolution. The encoder network doubles the number of channels, reducing the sample size of the feature map by half. The decoder network doubles the size of the feature map and half the channel numbers. Therefore, the encoder–decoder network transforms the input image into small-size and multichannel feature maps, and then decodes the feature map to an output image with the same size. At the same time, a skip-connection is adopted in the U-net network. This operation can connect feature maps in different sizes, which is helpful for gradient propagation and network convergence. All the convolutions in this neural network are followed by batch normalization (BN) and a rectified linear unit (ReLU) for faster training and to prevent the gradient vanishing problem.

**Figure 4:**
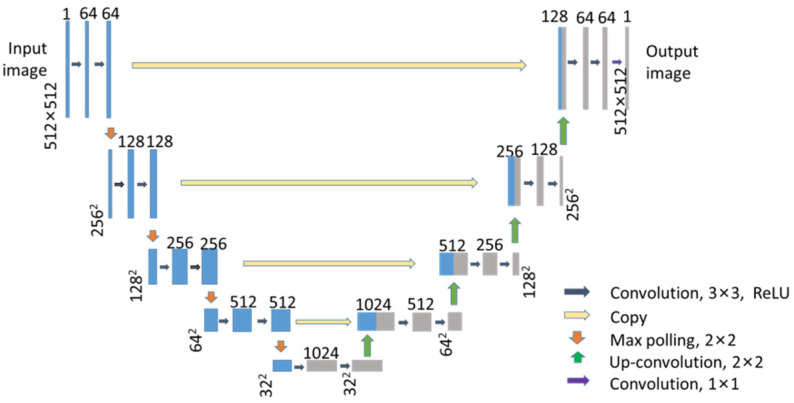
A-net network architecture. In the network framework, the blue box denotes different feature maps in different layers. The corresponding channel numbers are provided at the top of the box. The white box denotes copied feature maps. Different colored arrows represent different operations, which are labeled in the figure legend.

Since the sizes of the input image and output image of the U-net network are inconsistent, in order to make the output images of the U-net network have the same size as the input image, all valid convolution in the network is replaced with the same convolution. The employment of the same convolution makes the feature maps of the corresponding layers in encoding network and decoding network exactly the same size. Thereafter, it is appropriate to directly copy the feature map of the encoding network to the decoding network, as shown in Figure 4, and combine it with the feature map of the decoding network through the skip connection. This process avoids the crop operations in the U-net network, which can simplify the processing and reduce the image mismatching during cropping. Accordingly, the revised U-net network is named A-net in this paper.

The loss function of A-net is calculated by combining the cross-entropy loss function with a pixel-wise soft-max on the final feature map. The soft-max function can be calculated as follows:

(2) $$P_{i}\left(x\right)=\frac{\exp\left[a_{i}\left(x\right)\right]}{\sum_{j=1}^{M}\exp\left[a_{j}\left(x\right)\right]}$$

where $P_{i}\left(x\right)$ denotes the approximated maximum function, i represents the category of pixels, $a_{i}\left(x\right)$ represents the activation function score of the category of pixel is i with the pixel position x∈Ω and $\Omega \subset {\mathbb{Z}}^{2}$, M represents the number of classes,$\;a_{j}\left(x\right)$ represents the activation function score when the category of image pixel points is j, and $\sum_{j=1}^{M}\exp\left[a_{j}\left(x\right)\right]$ represents the sum of all classes of activation functions. In conclusion, $P_{i}\left(x\right)$ is the classification result of pixel x of class M, maximizing the most likely result while suppressing the probability of other categories. The sum of probabilities of all prediction categories is 1. For the i that has the maximum activation $a_{i}\left(x\right)$, the responding $P_{i}\left(x\right)\approx 1$, while for all the other i the responding $P_{i}\left(x\right)\approx 0$. Then, cross-entropy penalizes $P_{g\left(x\right)}\left(x\right)$ for a deviation from 1 at every position by Equation (3):

(3) $$E=\sum_{x\in \Omega }\omega \left(x\right)\log\left[P_{g\left(x\right)}\left(x\right)\right]$$

where ω∈Ω with Ω⊂ℝ denotes a weight and g(x) denotes the ground truth of each pixel. The purpose of setting ω is to give higher weights to pixels in the image that are close to the boundary points. In order to let the network learn to distinguish smaller boundaries, the weight graph is calculated in advance with ground truth of each pixel in the label images. 

### 3.4. Postprocessing

The A-net network predicts the input sub-images (512 × 512 pixels) of the test image and obtains corresponding predicted sub-images (512 × 512 pixels), which are subsequently assembled as prediction image (2048 × 2048 pixels). Then, a binarization step is applied on the prediction image in order to obtain a shrinkage outline of the microtubule structures. Subsequently, the binary image is multiplied with the test image to obtain the result image (shown in the Results section).

## 4. Experiments

### 4.1. SR_MUI Dataset

**Figure 5:**
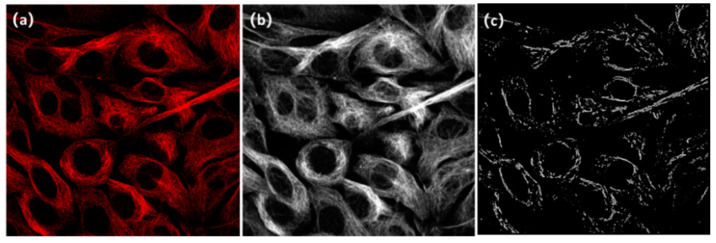
Images during the production of the SR_MUI dataset. (**a**) The raw 3t3 cell images captured by confocal microscope (Nikon A1 LFOV), the image size is 512 × 512; (**b**) the image obtained by the threshold denoising algorithm and three-dimensional Gaussian interpolation algorithm from (**a**), the image size is 2048 × 2048; (**c**) shows the high-resolution label images obtained by the DWDC algorithm and a binarization from (**a**), the image size is 2048 × 2048.

In this investigation, a biological microtubule image dataset, i.e., SR_MUI dataset, is constructed based on 3t3 cell images. The raw confocal image, the original image, and the high-resolution label images are shown in Figure 5.

**Figure 6:**
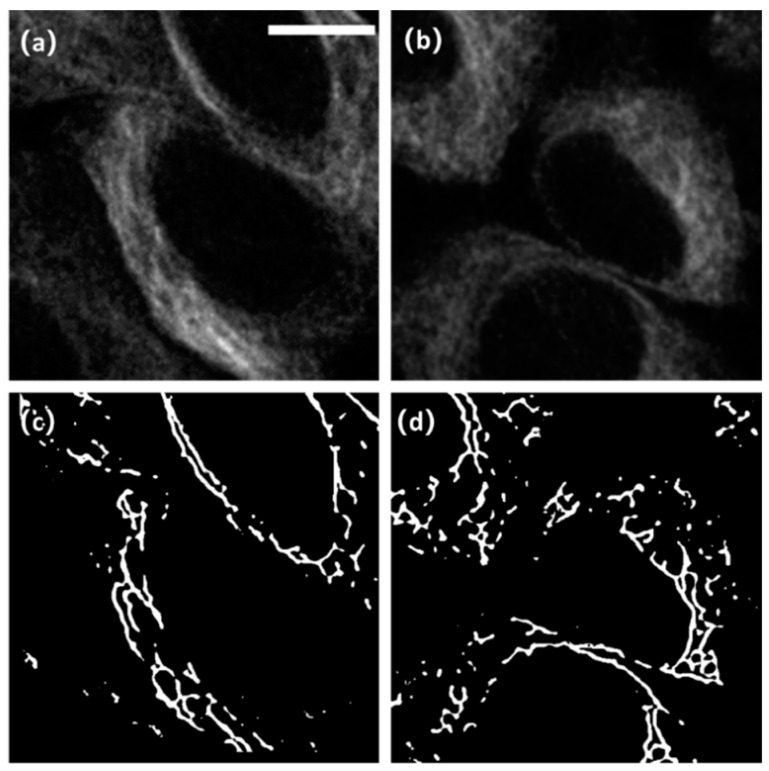
A preview of the SR_MUI dataset: (**a**,**b**) shows the sub-original images in the SR_MUI dataset and (**c**,**d**) show the corresponding high-resolution sub-label images. The sub-image of original and the sub-image of label compose image pairs to form the training dataset. The white scale bar represents 10 µm.

In the SR_MUI dataset, there are 200 image pairs for training and 50 images for testing. A preview of SR_MUI dataset is shown in Figure 6. It can be seen that the label images clearly extract the sketch of the microtubule structures from the noisy and blurry raw images.

### 4.2. Implementation

The numerical experiment is performed on the PyTorch platform with Python language. This study trained and tested the A-net network on a server with 10 NVidia RTX 2080TI GPUs. The epoch number is 200. The size of the minibatch is 1. In the entire training process, the A-net network adopts the Adam optimizer. In the testing process, the test images (2048 × 2048 pixels) were split into 16 sub-images (512 × 512 pixels). After input of the 16 sub-images into the A-net network to obtain the corresponding prediction images, the 16 prediction images were assembled to acquire the prediction image of 2048 × 2048 pixels. The resulting image is obtained after postprocessing.

## 5. Results

**Figure 7:**
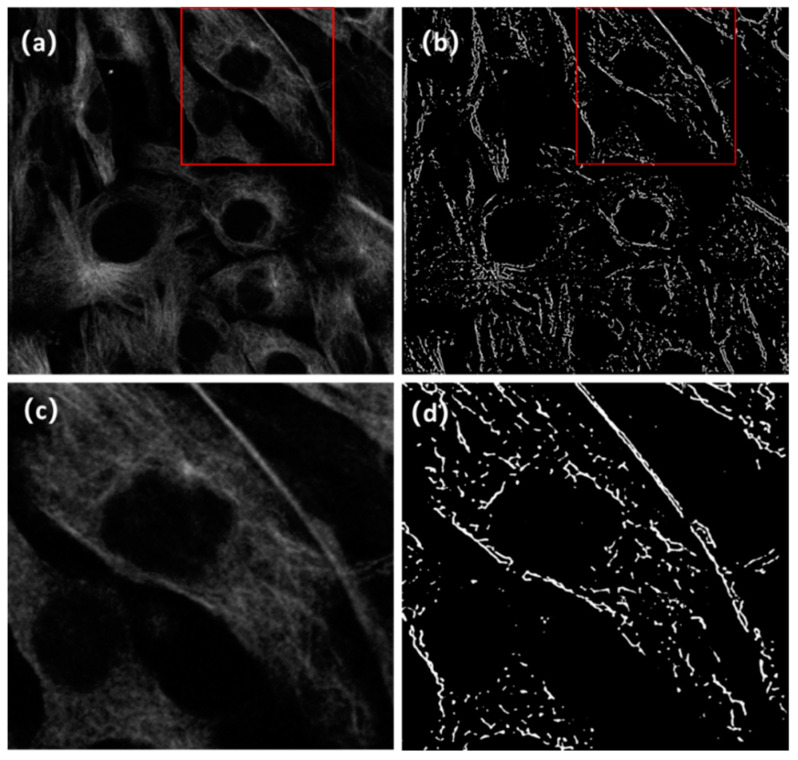
Comparison of the test image and the result image. (**a**) A test image placed into the A-net network and (**b**) the resulting super-resolution image. The test image is obtained through applying binarization on prediction images output from the AU-net network and multiplying the binary image with the test image. The image size is 2048 × 2048. The comparison of local regions in the red boxes of the test image and the result image are shown in (**c**,**d**).

Figure 7a is a typical test image that has low SNR and poor resolution. The cluster of microtubule structures can be roughly distinguished from the crowded backgrounds; the image cannot provide more accurate information on the microtubule distribution. In contrast, in the result images after A-net training, the noise is significantly suppressed and the microtubule structure information is extracted from the test image. In addition, after zooming in on the local image structures of both the test and result images, it can be seen that the two images (Figure 7c,d) show consistent structures of the microtubule.

**Figure 8:**
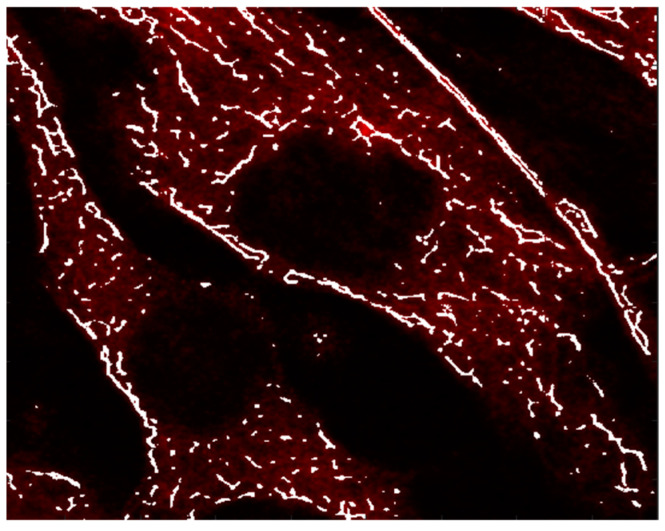
Test and result images are overlapped to show the consistency of structures. The test image is shown in red and the result image is shown in white. It can be seen that the main structures in the test picture have been extracted and show high consistency with the result image.

At the same time, in order to verify the consistency of microtubule structures between the test images and the result image, the two images are overlapped, as shown in Figure 8. For a different test image, the microtubule structures are concisely highlighted by the result image. The results clearly demonstrate the capability of the A-net network on preserving the raw filament-like structures. It should be noted, as a result of the high noise level, that some structures have been inevitably segmented. However, the results do not affect estimation of the overall topology of the microtubule structures.

**Figure 9:**
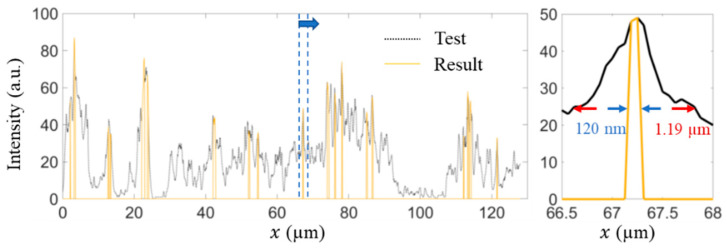
Comparison of the image intensity distribution between the test image and the result image. The left figure is the intensity profiles along the horizontal direction. On the right is a zoom-in view of the left figure in the marked position. The FWHM of the test image is 1.19 µm and that of the result image is 120 nm.

Figure 9 shows the comparison of the image intensity profiles along the horizontal direction between the test and result images. Here, only parts of the intensity profiles are plotted as an example to show the improvement in the images [48]. Observe that the image intensity distribution of the result image has a sharper peak and apparently lower noise. Overall, the result image retains a large amount of the structural information contained in the test image. The resolution of distinguishing the microtubule structures can be evaluated by the full width at half maxima (FWHM) [49,50]. As shown in the right column of Figure 9, the FWHM in the test image is 1.19 μm as compared to 120 nm in the result image. A super resolution with ~10 times improvement of resolution compared to the original image has been realized.

**Figure 10:**
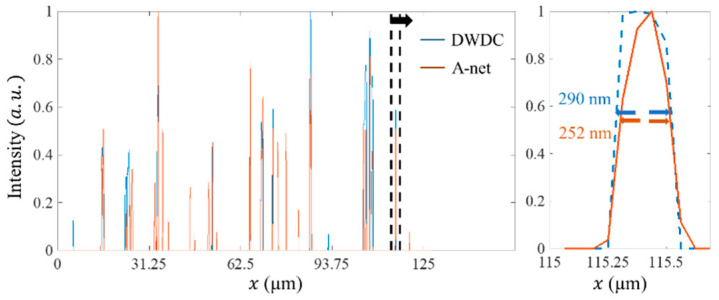
Comparison of the image intensity distribution between the result images from DWDC algorithm and A-net network. The left figure is the intensity profiles along the horizontal direction. On the right is a zoom-in view of the left figure in the marked position. The FWHM of the result image from the DWDC method is 290 nm, and that of A-net is 252 nm.

At the same time, the result images obtained by the A-net network are compared with those obtained by the DWDC algorithm, as shown in Figure 10. The FWHM of the result image obtained by the DWDC algorithm is 290 nm, and that of A-net network is 252 nm. An improvement of over 10% has been realized, even though DWDC has already exhibited super-resolution image processing capability.

For the result image, the SSIM and PSNR are 0.22 and 25.88, respectively, which are surprisingly low. This is because our purpose is to extract the information of microtubule structures with super resolution; the SSIM and PSNR values cannot provide effective evaluation on image processing. Although there is currently no appropriate criterion to evaluate the processing, the improvement in visual visibility and clarity of image structure is sufficient to demonstrate the effectiveness of this algorithm.

**Figure 11:**
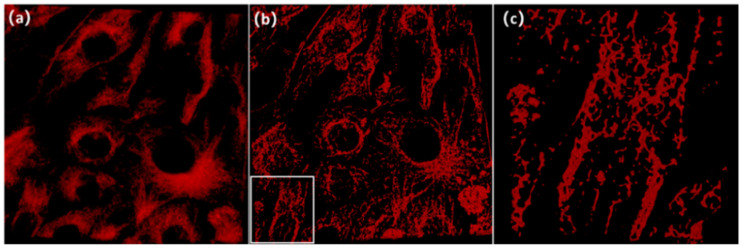
Three-dimensional microtubule structures reconstructed by a set of result images: (**a**) 3D microtubule structures by raw images (see Visualization 1 for details) and (**b**) 3D microtubule structures by the result image. (**c**) Zoom-in view of the lower left region of (**b**) (see Visualization 2 for details). The influence of noise has been significantly inhibited and the cell structure is clearly displayed.

Furthermore, in this study, the three-dimensional (3D) structures of microtubule on the basis of raw and result images layer-by-layer is built, as shown in Figure 11a,b, respectively. Figure 11c displays the 3D view of the lower left region of (b). As a result of the low signal–noise ratio of raw images, the 3D microtubule structures constructed from the raw images are blurry and unclear. The spatial distributions of the structures and even the skeleton are indistinguishable. In contrast, the 3D microtubule structures constructed from result images eliminate the noise and show the structures clearly. The major biological structures are continuous, which supports the effectiveness of the method.

## 6. Conclusions

In this investigation, a new method based on the A-net neural network and DWDC method is advanced and used to extract the molecular structure of 3t3 fibroblast microtubule networks from poor quality confocal images. The method requires a relatively small data set and avoids the difficulty of acquiring biological images in biomedical and medical imaging disciplines. The experimental results indicate a 10-fold improvement of spatial resolution, with a super resolution of 120 nm revealed from raw confocal images. The algorithm provides a general way for improving the resolution of filament-like structures with fewer computation resources. The algorithm will benefit broad biological and biomedical research, which rely strongly on optical imaging techniques.

## Data Availability

Not applicable.

## References

[1] Kim Y., Cha M., Choi Y., Joo H., Lee J. (2013). Electrokinetic separation of biomolecules through multiple nano-pores on membrane. Chem. Phys. Lett..

[2] Furuya K., Sokabe M., Furuya S. (2005). Characteristics of subepithelial fibroblasts as a mechano-sensor in the intestine: Cell-shape-dependent ATP release and P2Y1 signaling. J. Cell Sci..

[3] Hays J.B., Magar M.E., Zimm B.H. (1969). Persistence length of DNA. Biopolym. Orig. Res. Biomol..

[4] Friede R.L., Samorajski T. (1970). Axon caliber related to neurofilaments and microtubules in sciatic nerve fibers of rats and mice. Anat. Rec..

[5] Williams D.B., Carter C.B. (1996). The transmission electron microscope. Transmission Electron Microscopy.

[6] Crewe A.V., Isaacson M., Johnson D. (1969). A simple scanning electron microscope. Rev. Sci. Instrum..

[7] Adrian M., Dubochet J., Lepault J., McDowall A.W. (1984). Cryo-electron microscopy of viruses. Nat. Methods.

[8] Vicidomini G., Bianchini P., Diaspro A. (2018). STED super-resolved microscopy. Nat. Methods.

[9] Timpson P., McGhee E.J., Anderson K.I. (2011). Imaging molecular dynamics in vivo—From cell biology to animal models. J. Cell Sci..

[10] Radtke S., Adair J.E., Giese M.A., Chan Y.-Y., Norgaard Z.K., Enstrom M., Haworth K.G., Schefter L.E., Kiem H.-P. (2017). A distinct hematopoietic stem cell population for rapid multilineage engraftment in nonhuman primates. Sci. Transl. Med..

[11] Davila J.C., Cezar G.G., Thiede M., Strom S., Miki T., Trosko J. (2004). Use and application of stem cells in toxicology. Toxicol. Sci..

[12] Sousa A.A., Leapman R.D. (2012). Development and application of STEM for the biological sciences. Ultramicroscopy.

[13] Lu X., Wang Y., Fung S., Qing X. (2021). I-Nema: A Biological Image Dataset for Nematode Recognition. arXiv.

[14] Hunt B.R. (1995). Super-resolution of images: Algorithms, principles, performance. Int. J. Imaging Syst. Technol..

[15] Ng M.K., Bose N.K. (2003). Mathematical analysis of super-resolution methodology. IEEE Signal Processing Mag..

[16] Dong C., Loy C.C., He K., Tang X. (2014). Learning a deep convolutional network for image super-resolution. European Conference on Computer Vision.

[17] Dong C., Loy C.C., Tang X. (2016). Accelerating the super-resolution convolutional neural network. European Conference on Computer Vision.

[18] Ledig C., Theis L., Huszár F., Caballero J., Cunningham A., Acosta A., Aitken A., Tejani A., Totz J., Wang Z. Photo-Realistic Single Image Super-Resolution Using a Generative Adversarial Network. Proceedings of the IEEE Conference on Computer Vision and Pattern Recognition.

[19] Bai H., Bingchen C., Zhao T., Zhao W., Wang K., Zhang C., Bai J. (2021). Bioimage postprocessing based on discrete wavelet transform and Lucy-Richardson deconvolution (DWDC) methods. bioRxiv.

[20] Hojjatoleslami S.A., Avanaki M.R.N., Podoleanu A. (2013). Gh Image quality improvement in optical coherence tomography using Lucy–Richardson deconvolution algorithm. Appl. Opt..

[21] Devi A.G., Madhum T., Kishore K.L. (2015). A Novel Super Resolution Algorithm based on Fuzzy Bicubic Interpolation Algorithm. Int. J. Signal Processing Image Processing Pattern Recognit..

[22] Zhang Y., Fan Q., Bao F., Liu Y., Zhang C. (2018). Single-Image Super-Resolution Based on Rational Fractal Interpolation. IEEE Trans. Image Processing.

[23] Tao H., Tang X. (2003). Superresolution remote sensing image processing algorithm based on wavelet transform and interpolation. Image Processing Pattern Recognit. Remote Sens..

[24] Nitta K., Shogenji R., Miyatake S., Tanida J. (2006). Image reconstruction for thin observation module by bound optics by using the iterative backprojection method. Appl. Opt..

[25] Fan C., Wu C., Li G., Ma J. (2017). Projections onto Convex Sets Super-Resolution Reconstruction Based on Point Spread Function Estimation of Low-Resolution Remote Sensing Images. Sensors.

[26] Wang L.-G., Zhao Y. (2010). MAP based super-resolution method for hyperspectral imagery. Guang Pu Xue Yu Guang Pu Fen Xi=Guang Pu.

[27] Huang D., Huang W., Yuan Z., Lin Y., Zhang J., Zheng L. (2018). Image Super-Resolution Algorithm Based on an Improved Sparse Autoencoder. Information.

[28] Lin Z., He J., Tang X., Tang C.K. (2008). Limits of Learning-Based Superresolution Algorithms. Int. J. Comput. Vis..

[29] Rajaram S., Gupta M.D., Petrovic N., Huang T.S. (2006). Learning-Based Nonparametric Image Super-Resolution. EURASIP J. Adv. Signal Processing.

[30] Li X., Wu Y., Zhang W., Wang R., Hou F. (2019). Deep learning methods in real-time image super-resolution: A survey. J. Real-Time Image Processing.

[31] Sanchez-Beato A., Pajares G. (2008). Noniterative Interpolation-Based Super-Resolution Minimizing Aliasing in the Reconstructed Image. IEEE Trans. Image Processing.

[32] Zhou F., Yang W., Liao Q. (2012). Interpolation-Based Image Super-Resolution Using Multisurface Fitting. IEEE Trans. Image Processing.

[33] Zomet A., Rav-Acha A., Peleg S. Robust super-resolution. Proceedings of the 2001 IEEE Computer Society Conference on Computer Vision and Pattern Recognition, CVPR 2001.

[34] Patel V., Modi C.K., Paunwala C.N., Patnaik S. Hybrid Approach for Single Image Super Resolution Using ISEF and IBP. Proceedings of the 2011 International Conference on Communication Systems and Network Technologies.

[35] Lukeš T., Křížek P., Švindrych Z., Benda J., Ovesný M., Fliegel K., Klíma M., Hagen G.M. (2014). Three-dimensional super-resolution structured illumination microscopy with maximum a posteriori probability image estimation. Opt. Express.

[36] Babacan S.D., Molina R., Katsaggelos A.K. (2010). Variational Bayesian super resolution. IEEE Trans. Image Processing.

[37] Humblot F., Mohammad-Djafari A. (2006). Super-resolution Using Hidden Markov Model and Bayesian Detection Estimation Framework. EURASIP J. Adv. Signal Processing.

[38] Wu W., Liu Z., He X. (2011). Learning-based super resolution using kernel partial least squares. Image Vis. Comput..

[39] Gajjar P.P., Joshi M.V. (2010). New learning based super-resolution: Use of DWT and IGMRF prior. IEEE Trans. Image Processing.

[40] Bevilacqua M., Roumy A., Guillemot C., Alberi-Morel M.L. Low-complexity single-image super-resolution based on nonnegative neighbor embedding. Proceedings of the British Machine Vision Conference.

[41] Zhang Y., Du Y., Ling F., Fang S., Li X. (2014). Example-Based Super-Resolution Land Cover Mapping Using Support Vector Regression. IEEE J. Sel. Top. Appl. Earth Obs. Remote Sens..

[42] Ni K.S., Nguyen T.Q. (2007). Image Superresolution Using Support Vector Regression. IEEE Trans. Image Processing.

[43] Lu X., Yuan Y., Yan P. (2013). Image Super-Resolution Via Double Sparsity Regularized Manifold Learning. IEEE Trans. Circuits Syst. Video Technol..

[44] Yang J., Wright J., Huang T.S., Ma Y. (2010). Image Super-Resolution Via Sparse Representation. IEEE Trans. Image Processing.

[45] Zhu Z., Guo F., Yu H., Chen C. (2014). Fast Single Image Super-Resolution via Self-Example Learning and Sparse Representation. IEEE Trans. Multimed..

[46] Kim J., Kwon L.J., Mu L.K. Accurate Image Super-resolution Using Very Deep Nonvolutional Networks. Proceedings of the 2016 IEEE Conference on Computer Vision and Pattern Recognition (CVPR).

[47] Ronneberger O., Fischer P., Brox T. (2015). U-Net: Convolutional Networks for Biomedical Image Segmentation. International Conference on Medical Image Computing and Computer-Assisted Intervention.

[48] Vicidomini G., Hell S.W., Schönle A. (2009). Automatic deconvolution of 4Pi-microscopy data with arbitrary phase. Opt. Lett..

[49] Liu Y., Lu Y., Yang X., Zheng X., Wen S., Wang F., Vidal X., Zhao J., Liu D., Zhou Z. (2017). Amplified stimulated emission in upconversion nanoparticles for super-resolution nanoscopy. Nature.

[50] Westphal V., Rizzoli S.O., Lauterbach M.A., Kamin D., Jahn R., Hell S.W. (2008). Video-Rate Far-Field Optical Nanoscopy Dissects Synaptic Vesicle Movement. Science.

